# Gene encoding prolactin in cinnamon clownfish *Amphiprion melanopus* and its expression upon acclimation to low salinities

**DOI:** 10.1186/2046-9063-9-1

**Published:** 2013-01-01

**Authors:** Gyeong Eon Noh, Sum Rho, Yong Jin Chang, Byung Hwa Min, Jong-Myoung Kim

**Affiliations:** 1 Department of Fishery Biology, Pukyong National University, Busan 608-737, South Korea; 2 COREA, Center of Ornamental Reefs and Aquariums, Jeju 690-974, South Korea; 3 Aquaculture Industry Division, East Sea Fisheries Research Institute, Gangneung 210-861, South Korea

**Keywords:** Osmoregulation, Prolactin; Osmoregulation, Clownfish; *Amphiprion melanopus*

## Abstract

**Background:**

Prolactin (PRL) is a key hormone for osmoregulation in fish. Levels of PRL in the pituitary gland and plasma ion composition of clownfish seem to change to regulate their hydromineral balance during adaptation to waters of different salinities. In order to understand osmoregulatory mechanism and its association with growth performance and PRL in fish, the gene encoding PRL and its expression level in cinnamon clownfish *Amphiprion melanopus* upon acclimation to low salinity was analyzed.

**Results:**

The PRL gene of *A. melanopus* encoded a protein of 212 amino acid residues comprised of a putative signal peptide of 24 amino acids and a mature protein of 188 amino acids. Analysis of growth performance under different salinities of 34, 25, 15, and 10 ppt indicated that cinnamon clownfish could survive under salinities as low as 10 ppt. A higher rate of growth was observed at the lower salinities as compared to that of 34 ppt. Upon shifting the salinity of the surrounding water from 34 ppt to 15 ppt, the level of the PRL transcripts gradually increased to reach the peak level until 24 h of acclimation at 15 ppt, but decreased back as adaptation continued to 144 h. In contrast, levels of plasma Na^+^, Cl^-^, and osmolality decreased at the initial stage (4–8 h) of acclimation at 15 pt but increased back as adaptation continued till 144 h.

**Conclusion:**

Cinnamon clownfish could survive under salinities as low as 10 ppt. Upon shifting the salinity of the surrounding water from 34 ppt to 15 ppt, the level of the PRL transcripts gradually increased during the initial stage of acclimation but decreased back to the normal level as adaptation continued. An opposite pattern of changes - decrease at the beginning followed by an increase - in the levels of plasma Na^+^, Cl^-^, and osmolality was found upon acclimation to low salinity. The results suggest an involvement of PRL in the processes of osmoregulation and homeostasis in *A. melanopus*.

## Background

The marine ornamental industry has been continuously widening its proportion in the multi-billion dollar ornamental fish industry, including the associated equipments and accessories in recent years [1]. However, growth of the marine ornamental fish trade has been hindered by problems such as capture techniques damaging the ecosystem, over-exploitation, and high levels of mortality caused by inadequate handling and transport of sensitive living organisms [2]. One way to reduce the pressure on the ecosystem but to meet the increasing demands for marine ornamentals is to improve the efficiency of the capture as well as the culture system for desirable marine organisms. Therefore, it is necessary to understand the physiology of the target organisms. In teleosts, development and growth are known to be under the control of environmental factors such as temperature, salinity, and photoperiod [3]. There have been many reports about the effects of water salinity on the development and growth of fish [4]. Several advantages have been recognized for maintaining marine fish at lower salinities, probably by reducing osmotic stress and diseases associated with parasites which thrive higher salinities and by reducing the cost for maintaining the fish in a hatchery using artificial saltwater [5]. In addition, some euryhaline teleosts showed a better growth rate at intermediate salinity conditions, i.e. in brackish water of 8–20 ppt [3].

Cinnamon clownfish *A. melanopus* is one of the most popular species in marine ornamental trade. Clownfishes are protandric hermaphrodites, indicating that males change their sex to females. Most clownfish live in groups consisting of a female, a male, and several subadults and/or juveniles. The female is the most dominant member of the group, and her constant maintenance of the pecking order prevents the male from changing sex. Developments of mature sex organs as well as the growths of subadults are repressed by the presence of the adult pair. By maintaining a size disparity between members in the pecking order, lower-ranking clownfishes are able to reduce the conflict and are less likely to be evicted from the host anemone [6,7]. In addition, cinnamon clownfish are known to inhabit the regions of the lagoon and outer reef environments in the Great Barrier Reef of Australia, Indonesia, and the Solomon Islands, and in the tropical regions where freshwater input is pervasive throughout the wet tropics, at least in a seasonal context, from direct rainfall and river runoff. This indicates that clownfish have an ability to adapt to water with low salinity and are of interest for studying the physiology associated with social hierarchy and the osmoregulatory mechanism of fish.

Prolactin (PRL) is a key hormone important for osmoregulation in fish by regulating the entry of ions and water uptake. In general, levels of PRLs in the pituitary gland and plasma ion composition of fish seem to be increased during adaptation to freshwater for regulating the hydromineral balance by decreasing water uptake and increasing ion retention [8]. Since most of the low salinity adaptation studies in marine fish have been carried out with a rather large edible fish, it is necessary to examine the role of PRL in marine ornamental fish and the optimum salinity for its growth. For this, the gene encoding PRL and its transcript level, together with the growth performance of *A. melanopus*, were analyzed upon its adaptation to low salinity.

## Results

### Gene structure and amino acid sequence of PRL

**Figure 1:**
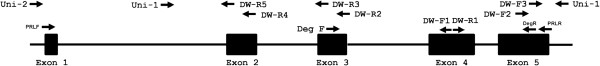
**Structure of gene encoding PRL of cinnamon clownfish *A. melanopus. ***Positions corresponding to the primers used for PCR amplification are presented by arrows. PCR primers Deg-F and Deg-R were designed from the conserved regions of prolactins reported from other species in Genbank. DW-F and DW-R indicate primers used for DNA walking to obtain a full-length prolactin gene. Exons are represented by dark boxes and introns are represented by lines.

**Figure 2:**
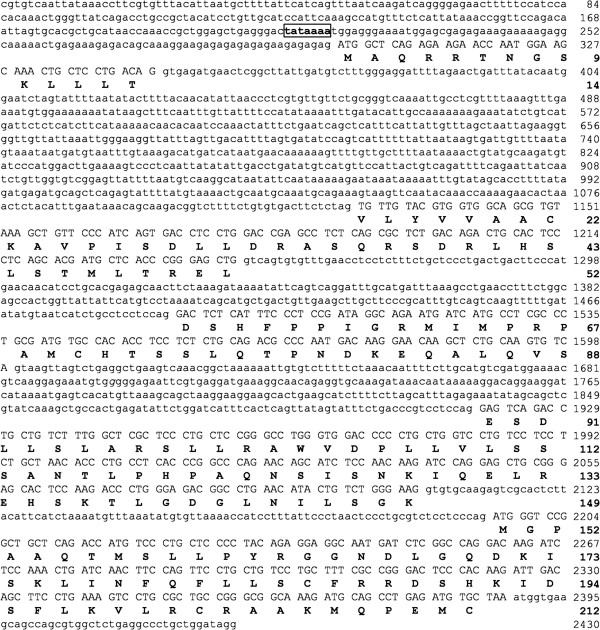
**PRL gene of *A. melanopus *and its coding sequence.** Coding regions in the exons are shown as upper cases and non-coding regions are shown as lower cases. The corresponding amino acid sequences are indicated as bold characters. The boxed sequence represents a putative TATA box.

The gene encoding PRL of cinnamon clownfish was obtained by PCR amplification using genomic DNAs and cDNA templates and primers as described (Table 1, Figure 1). The PRL gene consists of five exons (639 bp) separated by four introns (1,438 bp) and the sizes of the five exons were 43, 113, 108, 183, and 192 bp, respectively (Figure 2). An ORF encoded a protein of 212 aa residues comprised of a putative signal peptide of 24 aa and a mature protein of 188 aa. The sizes of the four introns were 784, 241, 320, and 93 bp, respectively. All the sequences found at the borders of introns contain consensus GT and AG splicing signals, and a TATA box (TATAAAA) was found at position −91. The PRL gene sequence was registered in the GenBank with an accession number HQ441171.

**Table 1:** Primers used for PCR amplification of prolactin genes

**Primers**	**Sequences (5′→3′)**
Deg-F	CCCTCCATGTGCCACACCTCC
Deg-R	AGGACTTTCAGGAAGCTGTCAAT
DW-F1	GAGCTGCAGGAGCACTCCAAGAC
DW-F2	CTCGGCCAGGACAAGATCTCCAA
DW-F3	TTCCTGCTGTCCTGCTTTCGCCG
DW-R1	GTCTTGGAGTGCTCCTGCAGCTC
DW-R2	TGACACTTGCAGAGCTTGTTCCTTGTC
DW-R3	CTTGTCATTGGGCGTCTGCAGAG
DW-R4	CTGATGGGAACAGCTTTACACGCTGC
PRLF	ATGGCTCAGAGAAGAACCAATGGAAGC
PRLR	TTAGCACATCTCAGGCTGCATCTTTGC

Deg: degenerate primer; DW: DNA walking; F: forward; PRL: prolactin; R: reverse.

### Alignment of amino acid sequence and phylogenetic analysis

**Figure 3:**
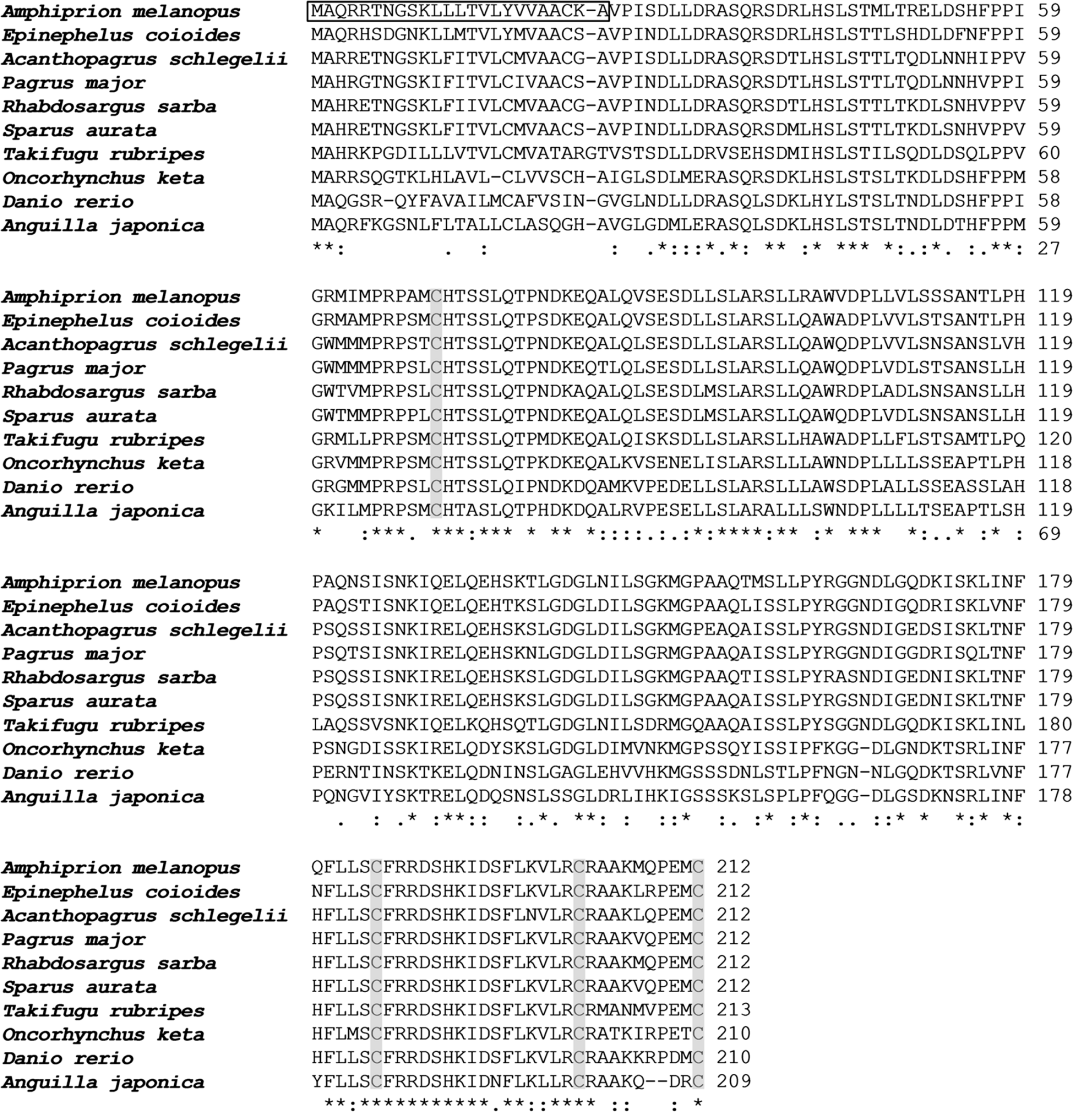
**Multiple alignment of amino acid sequences of PRL in *A. melanopus *(AEB00558) as compared to those of other teleosts.** Other prolactin sequences from *Epinephelus coioides* (AAO11695), *Acanthopagrus schlegeli* (AAX21764), *Pagrus major* (BAE43854), *Rhabdosargus sarba* (ABB17072), *Sparus aurata* (AAC26852), *Takifugu rubripes* (NP_001072092), *Oncorhynchus keta* (CAA45407), *Danio rerio* (NP_852102), and *Anguilla japonica* (AAO17792) were aligned by using ClustalW. The signal peptide sequence is marked by a box. Pairs of cysteine residues are shaded in grey. Identical amino acids among proteins are indicated by asterisks.

**Figure 4:**
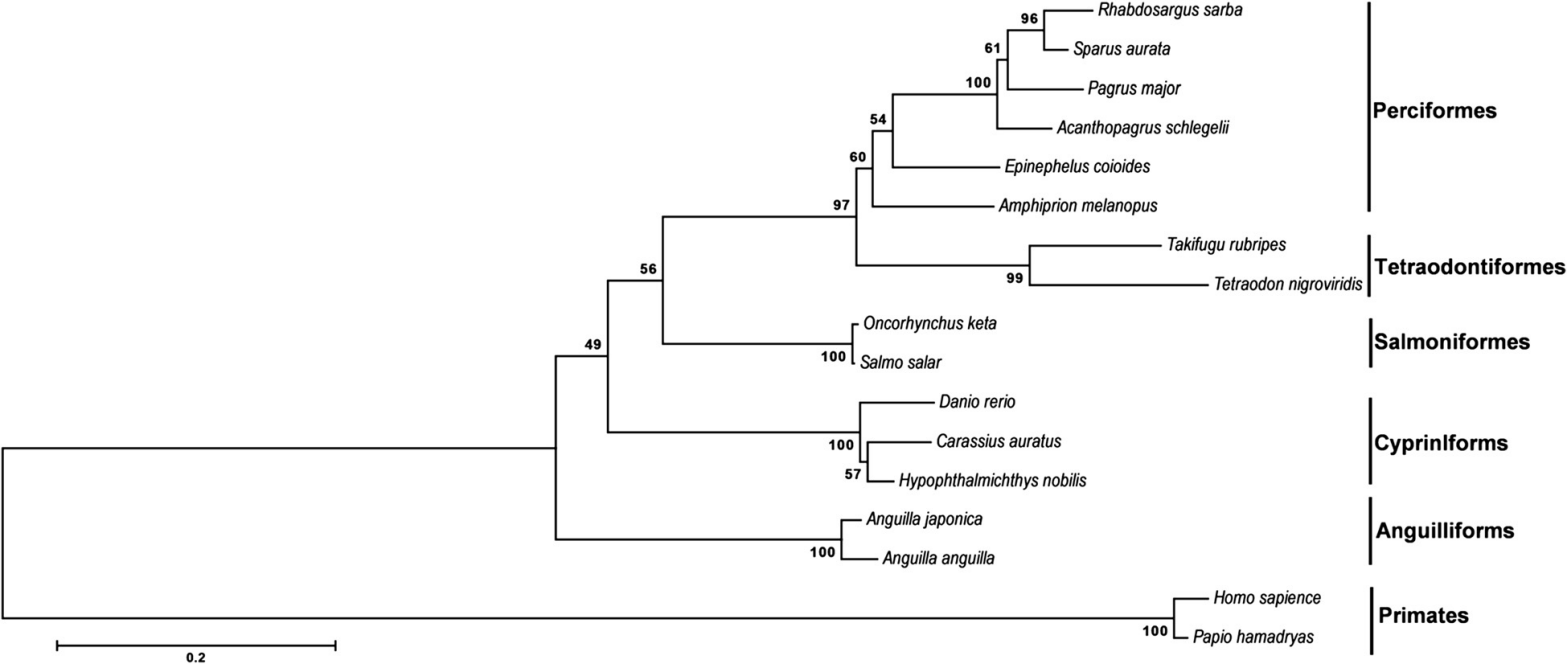
**Construction of neighbor-joining tree based on amino acid sequences of PRLs.** Bootstrap values are indicated for each node. Taxonomic groups are indicated on the right. Prolactin sequences from *A. melanopus* (AEB00558), *Epinephelus coioides* (AAO11695), *Acanthopagrus schlegeli* (AAX21764), *Pagrus major* (BAE43854), *Rhabdosargus sarba* (ABB17072), *Sparus aurata* (AAC26852), *Takifugu rubripes* (NP_001072092), *Oncorhynchus keta* (CAA45407), *Danio rerio* (NP_852102), *Anguilla japonica* (AAO17792), *Tetraodon nigroviridis* (AAR25696), *Salmo salar* (NP_001117140), *Carassius auratus* (AAT74865). *Hypophthalmichthys nobilis* (CAA43383), and *Anguilla anguilla* (CAA48902) were used for comparison. Prolactin sequences from *Papio hamadryas* (ADG56475) and *Homo sapiens* (AAH88370) were included as outgroups.

Amino acid sequence comparison of *A. melanopus* PRL with those of other teleosts (Figure 3) using ClustalW [9] indicated aa identities of 83% with *Epinephelus coioides* (Accession No. AAO11695), 79% with *Acanthopagrus schlegeli* (AAX21764), 78% with *Pagrus major* (BAE43854), *Rhabdosargus sarba* (ABB17072) and *Sparus aurata* (AAC26852), 72% with *Takifugu rubripes* (NP_001072092), 69% with *Oncorhynchus keta* (CAA45407), 59% with *Danio rerio* (NP_852102), and 58% with *Anguilla japonica* (AAO17792). Cinnamon clownfish PRL contains four cysteine residues in the loci conserved in the PRLs of other teleosts. A phylogenetic tree also indicated a similarity between cinnamon clownfish and other teleost but a broad distinction from non-teleost PRLs (Figure 4).

### Growth of cinnamon clownfish reared under different salinity conditions

**Figure 5:**
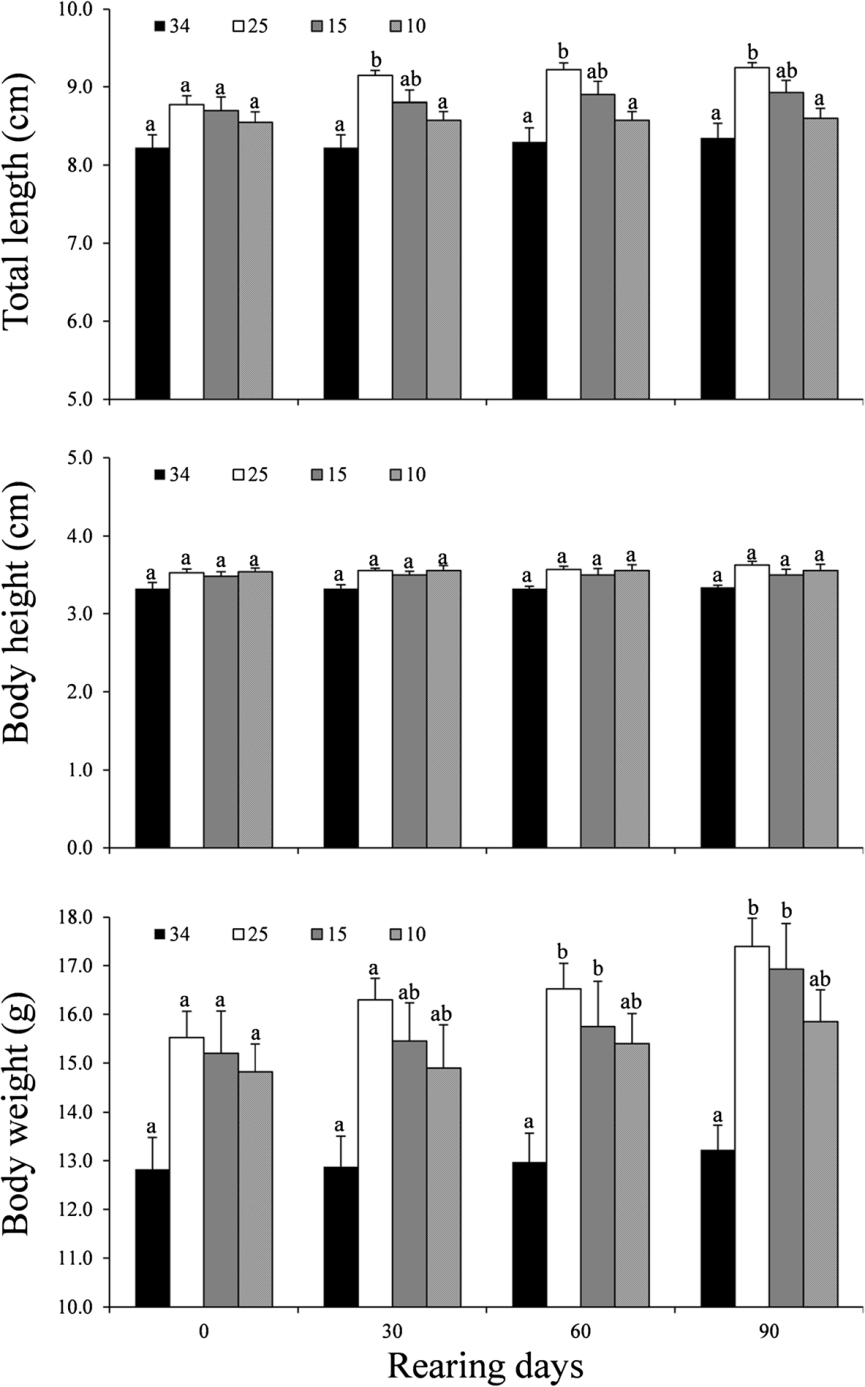
**Effects of salinity on total length, body height, and body weight of the two dominant *A. melanopus *species in the group reared at different salinities for 90 days: 34, 25, 15, and 10 ppts.** Values are means±SD (n=4) and different letters indicate significant differences (P<0.05).

Survival rates and the growth performance of *A. melanopus* were tested under different salinities. The survival rates of cinnamon clown fish grown under salinities of 34, 25, 15, and 10 ppt were 86.7, 58.8, 43.8, and 52.9%, respectively, although the values seemed to be influenced by the attacking behavior of the dominant clownfish. Effects of salinity on growth were analyzed by the total length (TL), body height (BH), and body weight (BW) of two dominant fishes with larger sizes, as the attacking behavior in the group might have had a greater influence on the growth of the smaller fish. The result showed a significantly higher TL and BW of clownfish reared at 25 ppt as compared to those grown at 34 ppt for 90 days (Figure 5). Clownfish groups acclimated at lower salinities of 15 ppt and 10 ppt also showed a higher BW as compared to that of 34 ppt.

### Expression of PRL in cinnamon clownfish upon acclimation to low salinity

**Figure 6:**
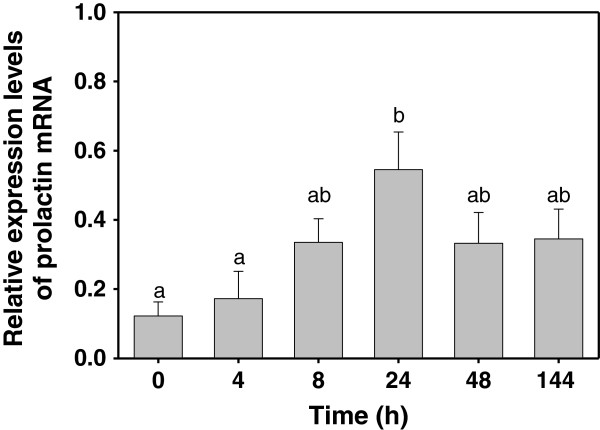
**Analysis of PRL mRNA level in cinnamon clownfish *A. melanopus *upon exposure to 15 ppt.** Total RNA(0.5μg) prepared from pituitary gland was used for generating cDNA by reverse transcription followed by PCR amplification using PRL-specific primers. Levels of β-actin mRNA were evaluated as a control. The expression level of each prolactin mRNA was normalized with respect to the level of β-actin transcript. Each value represents the mean±SE (n=4) and the same letters indicate no significant difference (P>0.05).

In order to analyze the level of PRL expression in cinnamon clownfish, RNAs were isolated from the pituitary glands of the fish reared in water where the salinity was shifted from 34 ppt to 15 ppt. Fish were collected at 0, 4, 8, 24, 48, and 144 h of acclimation at 15 ppt and subject to RNA isolation followed by a PRL transcript analysis using RT-PCR (Figure 6). The result showed an increase in the PRL transcript reaching the highest level (almost 5-fold) at 24 h of acclimation at 15 ppt followed by a decrease in the level, but it was still higher than that of 34 ppt, as adaptation continued to 48 h and 144 h.

### Plasma parameters

The levels of Na^+^, Cl^-^, and osmolality were measured from the plasma of the fish exposed to 15 ppt. Levels of Na^+ ^in the plasma were 205±3.9, 172±3.8, 170±3.9, and 175±3.3 mEq/L, respectively, at 0, 4, 8, and 24 h of acclimation (Table 2). The level was increased to 192±2.2 mEq/L as acclimation extended to 144 h. The levels of Cl^- ^were 189.1±1.0, 159.6±3.6, 156.2±4.2, 158.2±2.2, and 174.1±2.1 mEq/L at 0, 4, 8, 24, and 144 h, respectively, of adaptation at 15 ppt. A similar tendency of plasma osmolality to decline in the initial stage but to recover its level as acclimation continued was also observed, as indicated by 379±1.0, 312.5±5.5, 313.0±3.0, 331.5±2.5, and 351.5±0.5 mOsm/kg of osmolalities at 0, 4, 8, 24, and 144 h, respectively, of adaptations (Table 2).

**Table 2:** **Plasma Na**^**+**^**, Cl**^**- **^**ions, and osmolality in *A. melanopus *upon exposure to 15 ppt water**

**Time (h)**	**Na**^**+ **^**(mEq/L)**	**Cl**^**- **^**(mEq/L)**	**Osmolality (mOsm/kg)**
0	205 ± 3.9	189.1 ± 1.0	379.0 ± 1.0
4	172 ± 3.8	159.6 ± 3.6	312.5 ± 5.5
8	170 ± 3.9	156.2 ± 4.2	313.0 ± 3.0
24	175 ± 3.3	158.2 ± 2.2	331.5 ± 2.5
144	192 ± 2.2	174.1 ± 2.1	351.5 ± 0.5

Fish were transferred from 34 ppt (0 h) to 15 ppt water. Values shown as mean±S.E. (n=6) were measured from fish reared at 15 ppt for the times indicated.

## Discussion

Clownfish have an ability to survive against a range of salinity changes as they are found in tropical habitats where freshwater input is pervasive in a seasonal context. The protandric hermaphrodites live as a social unit in which dominance affects the development and growth of the subordinate members. The study focused on the effects of salinity on the development and growth of the cinnamon clownfish, one of the most demanded species in the marine ornamental industry. Growth analysis of cinnamon clownfish at salinities of 34 ppt, 25 ppt, 15 ppt, and 10 ppt indicated that *A. melanopus* could survive at salinities as low as 10 ppt over a period of 90 days. The result is similar to that of skunk clownfish *A. akallopisos* with high survival rates under the condition of salinities up to 53 ppt and tolerance up to 6 ppt of salinity [10]. Lower survival rates of *A. melanopus* at lower salinities were inconsistent with the previous studies examining the relationship between growth and salinity in juvenile black bream [11]. This might be due to their social organization in cinnamon clownfish because the growth parameters at lower salinities were higher than those at 34 ppt meaning a better growth, although we could not exclude the possibility of lower density effects on the growth. In addition, the results seemed to be affected, at least partly, by the social hierarchy system in cinnamon clownfish as it was often observed that the one (or two) dominant fish in the tank bit the smaller fish to death or chased them during the experiment. In fact, most of the dead fish were the small in size and were found to have serious wounds on their bodies and fins. Therefore, it is plausible to imagine that the stressful condition due to a decrease in salinity might contribute to more attacking behaviors leading to the decline of survival rate and growth [12].

According to Wilkerson [6], a typical clownfish social unit consists of a mature female, a mature male, and often with a few adolescents. A size-based, hierarchic dominance was noticed in common clownfish *A. ocellaris*: the female is the largest (rank 1) followed by the second largest male (rank 2) and the non-breeders get progressively smaller as their hierarchies are descended to ranks 3–6. Dominants seemed to gain no benefits from subordinates which are potential challengers for their position and occasionally evict or kill the subordinates of similar sizes. The small fish avoids the chases and challenges of larger members by signaling its acceptance of their positions in the social unit. An aggressor’s behavior and sound production involve chasing with chirping, jaw popping, ventral leaning, and dorsal leaning, following the response of an appeaser’s behavior with rapid body quivering as the individual drifts upward with head up or down, clicking sounds, convulsive body shaking, quivering, and ventral leaning. All the behavioral patterns above were observed during our experiments with immature fish with size differences. It was also occasionally observed that the larger one would chase the smaller one and the latter would just swim away and fail to take position as a member. We conjectured that competition and even the attacking of smaller fish, which occurred during the process of stabilizing a hierarchy in the group under different salinities, might influence the survival rate. It will be interesting to examine the effect of low salinity on the growth of juvenile stages which showed schooling behavior without attacking behavior.

Analysis of growth performance at different salinities was carried out with two dominant fishes in the group to avoid the influence of dominance-related behavior, such as attacking the smaller fish. A higher rate of growth was observed in the groups reared at lower salinities as compared to those grown at 34 ppt. The result was consistent with other studies showing a better growth at intermediary salinity conditions, i.e. a brackish water of 8–20 ppt, in fish including *O. niloticus*, *Mugil* sp., *Anarhichas lupus*, *S. aurata*, and *Hippoglossus hippoglossus* L [13-16]. According to Bœuf and Payan [3], this seemed to be attained by several factors including i) decline of energy cost: a drastic reduction of metabolic rate was found in rainbow trout *O. mykiss* at isotonic water [17] and the lowest gill Na^+^, K^+^-ATPase in juvenile turbot *S. maximus* correlate well with the best growth at intermediate salinity [18], ii) food intake and/or food conversion: better growth rates in cod *Gadus morhua* at lower salinity correlated well with an increase in food conversion efficiency [19] although more complex factors such as temperature and fish species might also be involved [16], and iii) hormones: thyroid hormone, growth hormone, and insulin-like growth factor were known to be involved in osmoregulation upon temporary salinity increase in salmonids [20]. Our results showed an increase in the level of PRL during an early stage of acclimation followed by a decrease as acclimation continued (see below). The results indicated that PRL may not be a major factor responsible for the higher growth of cinnamon clownfish in the intermediary salt condition during a long-term growth trial. At present, the plausible hypothesis suggests that the energy to retain osmotic pressure is saved in isotonic water and could be transferred for growth although clearer evidence is necessary.

PRL belongs to the same family as growth hormones, mammalian placental lactogen, and teleostean somatolactin and, in teleost, is known to act as a freshwater-adapting hormone [8]. The structure of the PRL gene consisting of five exons and four introns was consistent with PRLs reported from other fish [21,22]. While nonteleosts such as Russian sturgeon *Acipenser gueldenstaedtii* and marbled lungfish *Protopterus aethiopicus* have mature PRLs of 204 aa and 200 aa, respectively [23,24], teleosts including European eel *Anguila anguila*, common carp *Cyprinus carpio*, and goldfish *Carassius auratus*, have PRLs of the sizes between 185 and 188 aa [25-28]. In addition, long (188 aa; PRL I) and short (177 aa; PRL II) forms of PRLs were reported in common carp and tilapia [8,27,29]. The PRL gene of *A. melanopus* obtained in this study encoded a prepeptide of 212 aa including a signal peptide of 24 aa and a mature protein of 188 aa, as similar to other piscine PRLs synthesized as prohormones with signal peptides of 23–24 aa [8]. It also contains four cysteine residues (Figure 3) which might form two disulfide bridges in the mature protein as in other teleost PRLs [30], in contrast to nonteleost and mammalian PRLs having three disulfide bridges. Therefore, teleost PRLs can be characterized by lacking a disulfide bond in the N-terminal region and their lengths are shorter (by an absence of 12–14 aa at the N-terminus) than those of nonteleost and mammalian. Absence of the N-terminal disulfide bridge might be important for osmoregulation as shown by the fact that injection of ovine PRL with a disrupted N-terminal disulfide bond increased the bladder potency in stickleback *Gillichthys *[31].

Salinity change in the surrounding waters cause an imbalance of ions and water in the fish [32]. Therefore, fish need to release ions and absorb water in seawater but absorb ions and release water in freshwater to maintain a constant level of osmolality. While *A. melanopus* could survive at salinities as low as 10 ppt, the fish consumed feed well at 15 ppt as similar to that in 34 ppt (Noh & Kim, personal observation). Therefore, experiments for examining the correlation of PRL and osmoregulation were carried out at 15 ppt, which is similar to the salinity of the brackish water where cinnamon clownfish were found in nature. Upon shifting to 15 ppt, the level of PRL transcripts in the pituitary was increased until 24 h of adaptation and then declined slowly as acclimation extended to 48 and 144 h. This was consistent with other studies showing an increase of the PRL transcript at lower salinity in pufferfish *T. rubripes*, black porgy *A. schlegeli*, gilthead sea bream *S. auratus*, and Mozambique tilapia *O. mossambicus *[30,33-35]. PRL levels in Atlantic salmon parr *Salmo salar* and ayu *Plecoglossus altivelis* were decreased upon transferring from freshwater to seawater [36,37]. It was also noticed that PRL influenced the retention and uptake of Na^+^ in fish adapting to freshwater. An opposite pattern of changes, decrease at the beginning of salinity acclimation but increase to the normal level as adaptation continued, was found in the plasma levels of Na^+^, Cl^-^, and osmolality. Correlation between the induction of the PRL transcript and osmolality changes upon exposure to low salinity indicates that PRL is involved during the early stage of adaptation to low salinity in cinnamon clownfish.

## Conclusions

PRL gene in cinnamon clownfish consists of five exons encoding an ORF with a putative signal peptide of 24 aa and a mature protein of 188 aa as similar to those of euryhaline fish. PRL is involved in the initial stage of low salinity acclimation in cinnamon clownfish as shown by reaching a peak level at 24 h followed by a decrease in the level, indicating homeostasis. Cinnamon clownfish can be grown at salinities as low as 10 ppt. A better growth condition could be obtained at a lower salinity, providing the dominance-related killing of the smaller fish is prohibited. These are useful findings for marine ornamental fish aquaculture.

## Methods

### Animals and sampling procedure

Rearing experiments for PRL transcript analysis were carried out during June 2011. Fish (n=30, 67.3±7.9 mm total length and 7.3±2.5 g body weight) were divided into six groups and acclimated in a 120 L circular tank with a recirculatory system (34 ppt; 13 light: 11 dark cycle; 26.5±1.0°C) for 5 days. Fish were fed Formula-one marine pellet (Ocean Nutrition, USA) twice a day. The salinity of the seawater (SW, 34 ppt) was lowered immediately by adding underground water until it reached 15 ppt. Five fish were collected with different intervals for 0, 4, 8, 24, 48, and 144 h, respectively, of acclimation. Fish were killed by spinal transection for the collection of the pituitary gland. Immediately after collection, the pituitary sample was frozen in liquid nitrogen and stored at −80°C until RNA isolation for RT-PCR.

Growth of cinnamon clownfish (n=7~8) under different salinities was carried out in 120 L tanks (26.5±1.0°C, 13 h light: 11 h dark) from July till the end of 2011. Fish were reared in the water salinities of 34, 25, 15, and 10 ppts for 90 days and their survival rate, total length (TL), body height (BH), and body weight (BW) were estimated every 30 days.

### Cloning and characterization of the PRL gene in cinnamon clownfish

Genomic DNA was extracted from the whole blood of clownfish using a AccuPrep® Genomic DNA extraction Kit (Bioneer Inc., Korea). PCR primers were designed from the conserved regions of PRLs reported in GenBank (Table 1). PCR was carried out in 20 μL mixture containing genomic DNA (0.05 μg/μL), Deg F, and Deg R primers (1 μM), and 1x HiQ-PCR Mix (GenoTech, Korea). Each reaction was carried out with an initial denaturation at 94°C for 4 min, 30 cycles of reactions composed of denaturation at 94°C for 30 s, annealing at 60°C for 30 s, and extension at 72°C for 50 s, followed by a final extension at 72°C for 5 min. DNA walking (Seegene, Korea) was performed according to the manufacturer’s instructions (Figure 1) with DW-primers to obtain the regions corresponding to the 5′- and 3′-ends of the PRL gene. PCR products were purified using a gel extraction kit (NucleoGen, Korea) and cloned into Topcloner°TA kit (Enzynomics, Korea) according to the manufacturer’s protocol.

### Expression of the PRL gene in cinnamon clownfish by reverse transcription (RT)-PCR

Total RNA was extracted from pituitary glands (n=4) using TRI Reagent (Sigma, USA). The total RNA sample was treated with DNase I for 30 min at 37°C. First-strand cDNA was synthesized in 20 μL reaction containing 0.5 μg total RNA and 0.5 μM dT_15_, ImProm-II^™ ^Reverse Transcriptase (Promega, USA), 6mM MgCl_2_, 0.5mM dNTPs, and 20 unit RNase Inhibitor (Enzynomics, Korea). RT-PCR was carried out with cDNA templates and PRLF/R primers at 94°C for 4 min, and 20 cycles consisting of denaturation at 94°C for 30 s, annealing at 60°C for 30 s, and extension at 72°C for 50 s, followed by a final polymerization at 72°C for 5 min. Levels of β-actin mRNA were evaluated to normalize the expression level of prolactin mRNA as described [38]. PCR products were resolved on agarose gel electrophoresis followed by staining with ethidium bromide and quantification by Gel Doc System/Station (BIORAD, USA).

### Plasma parameters analysis

Blood was pooled from the caudal vasculature of five fish using a 1 mL syringe coated with heparin (25,000 IU/5 mL). Plasma samples were separated by centrifugation and stored at −80°C until analysis. Levels of plasma Na^+ ^and Cl^− ^ions were analyzed by using a Biochemistry Auto Analyzer (model 7180; Hitachi, Tokyo, Japan). Plasma osmolality was measured by using a Vapor Pressure Osmometer (Vapro 5520; Wescor Co., Logan, UT, USA).

### Sequence comparison

The amino acid sequence of cinnamon clownfish PRL was aligned with the other PRL using ClustalW [9]. The phylogenetic tree was constructed by the neighbor-joining method using MEGA v 5.0 with PRLs of various species selected from NCBI. The reliability of the phylogenetic tree was assessed by using a bootstrap with 1000 replicates with outgroups.

### Statistics

Analysis was carried out with the SPSS statistical package (SPSS ver. 20, USA). One-way ANOVA followed by a post hoc multiple comparison test (Tukey’s test) was used to compare differences in the groups. A non-parametric Kruskal-Wallis test followed by a Tukey’s test using ranks was used for analysis of data collected from the two largest fish in the group and comparison of the PRL gene expression.

## Abbreviations

PRL: Prolactin; aa: Amino acid.

## Competing interest

The authors declare that they have no competing interests.

## Authors’ contributions

GE carried out gene cloning/growth analysis experiments and drafted the manuscript. SR provided clownfish and involved in growth experiment. YC contributed to design the experiment and data analysis. BM carried out osmolality analysis. JK contributed to experimental design, data analysis, and manuscript preparation. All authors read and approved the final manuscript.

## Authors’ information

GEN an expert in the field of fish physiology.

SR is a CEO of *CCORA* and an expert in the field of marine ornamental industry.

YJC and BHM are experts in the field of fish physiology.

JMK is a biochemist and molecular biologist.
